# The Effect of *Perilla frutescens* Extract on the Oxidative Stability of Model Food Emulsions

**DOI:** 10.3390/antiox3010038

**Published:** 2014-01-22

**Authors:** Monika Skowyra, Victor Falguera, Nurul A. M. Azman, Francisco Segovia, Maria P. Almajano

**Affiliations:** 1 Chemical Engineering Department, Technical University of Catalonia, Av. Diagonal, 647, Barcelona 08028, Spain; E-Mails: skowyra.monika@gmail.com (M.S.); aini.azman@gmail.com (N.A.M.A.); segoviafj@gmail.com (F.S.); 2 Agricultural Knowledge & Innovation Services (AKIS International), Av. Dr. Robert, 33, Albatàrrec 25171, Spain; E-Mail: v.falguera@akisinternational.com

**Keywords:** perilla, polyphenols, antioxidants, oil-in-water emulsions, lipid oxidation

## Abstract

The polyphenolic profile of leaves and stalks of *Perilla frutescens*, was assessed as a source of natural antioxidants. The amount of caffeic and rosmarinic acids, determined by high-performance liquid chromatography (HPLC), were 0.51 mg/g dry weight (DW) and 2.29 mg/g DW, respectively. The measurement of scavenging capacity against the 2,2′-azino-bis-3-ethylbenzothiazoline-6-sulphonic acid (ABTS) radical cation, the oxygen radical absorbance capacity (ORAC), and the ferric reducing antioxidant power (FRAP) were 65.03 mg Trolox equivalents (TE)/g DW, 179.60 mg TE/g DW and 44.46 mg TE/g DW, respectively. *P. frutescens* extracts also showed good antioxidant properties in 10% sunflower oil-in-water emulsions during storage at 32 °C. Perilla extract at 320 ppm was as effective as butylated hydroxyanisole (BHA) at 20 ppm in slowing down the formation of hydroperoxides as measured by peroxide value, thiobarbituric acid reactive substances and hexanal content. The results of this study indicate that extract of *P. frutescens* may be suitable for use in the food matrix to help achieve potential health benefits.

## 1. Introduction

In most foodstuffs, lipid oxidation is a severe problem that causes rancid odors and flavors, modifies texture and color, and decreases shelf life [1]. These changes degrade functional and nutritional compounds of food, damage essential fatty acids, and produce oxidized polymers, which could raise safety concerns. Especially, this process is favored in oil-in-water emulsions due to the large contact surface between the oxidizable lipid hydroperoxides in emulsion droplets and water-soluble prooxidants resulting in the propagation of oxidation reactions [2]. To avoid this problem, synthetic antioxidants are commonly used, such as butylated hydroxytoluene and butylated hydroxyanisole [3]. However, in recent years, consumers have become increasingly concerned about the impact of food and food ingredients on their own health and this attitude has caused several changes throughout the food industry [4]. As part of these changes, food companies have been forced to seek for natural-origin counterparts for a number of ingredients that fulfill technological functions [5].

Plant extracts rich in phenolic compounds may be a good alternative to synthetic antioxidants to prevent lipid oxidation. Plants produce phenolic compounds to deal with reactive oxygen species and free radicals produced during photosynthesis. Inside the plant structure, lipid peroxidation has been known to associate with tissue injuries and disease conditions. Plant phenolic compounds can act as protective factors delaying the onset of lipid oxidation and decomposition of hydroperoxides in living tissues [6]. Therefore, such compounds are expected to play a similar role inside food matrices, provided that a suitable extraction and conservation method is found and optimized. In the process of testing new plant extracts as antioxidants, two major difficulties arise. On the one hand, there are several factors in the extract-obtaining process that must be analyzed, the most important of which are the method and the solvent used. These factors will determine the antioxidant content and activity of the resulting mixtures [7]. On the other hand, very often the results obtained when studying the isolated products using the *in vitro* tests correlate poorly with their ability to avoid oxidative impairment of foods [8,9]. This lack of equivalence is the consequence of complex interactions among the components of the food matrix, which may limit or enhance the activity of the tested extract. Therefore, functionality tests must be performed in model foods that consider the most important conditions of the actual food in which they are to be applied. In this way, oil-in-water emulsions have become a standardized model food to test the protective effect of antioxidant products against lipid oxidation [10]. Phenolic extracts of certain plant materials have been shown to neutralize free radicals in model systems. Widely used culinary herbs of the *Lamiaceae* family, such as rosemary, thyme, marjoram, and oregano, have gained the interest of many research groups [11,12,13,14].

*Perilla frutescens* (*Lamiaceae* family) is a traditional Chinese medicinal plant that is commonly used for a variety of diseases such as depression, inflammation, bacterial and fungal infections, allergy, intoxication, some intestinal disorders, and even tumors [15,16]. In Asian countries, such as Japan, Korea, and China, its leaves are commonly added to sushi, garnishes, and soups, and young raw leaves are often used to wrap cooked food. Its health-promoting effects have been mainly attributed to its content of phenolic acids (e.g., rosmarinic acid), flavonoids, and triterpenoids [17,18]. These components provide the extracts of this plant with proved antioxidant, anti-inflammatory, antibiotic, and antipyretic properties. Moreover, *Perilla frutescens* seed oil (known as PFSO) has also been shown to be a rich source of unsaturated fatty acids, especially omega-3 linolenic acid [19]. Although not yet reported in the literature, perilla extracts, being a rich source of various phenolic compounds, could therefore be incorporated in model emulsions as a source of natural antioxidant to prolong quality and stability.

The aim of this paper is to report a study of the antioxidant properties of purple perilla (*Perilla frutescens*) extracts in model emulsions stored for long periods, which can be representative of real food systems and their expected shelf life. Lipid oxidation was determined by following the formation of peroxide values as the primary oxidation products and thiobarbituric acid reactive substances and hexanal content as the secondary products. In addition, the content of the main phenolic compounds in perilla cultivated in Spain has been quantified.

## 2. Experimental Section

### 2.1. Raw Material

Purple perilla was grown in a greenhouse (Balaguer, Spain). Stalks and leaves of perilla were collected, dried, and ground to a homogenous powder in collaboration with the company, Pàmies Hortícoles. The powder was stored in darkness, at room temperature until extraction. Refined sunflower oil was purchased in a local market.

### 2.2. Reagents

6-Hydroxy-2,5,7,8-tetramethylchromane-2-carboxylic acid (Trolox), gallic acid, rosmarinic acid, caffeic acid, phosphate buffered saline (PBS), 2,2′-azino-bis-(3-ethylbenzothiazoline)-6-sulfonic acid diammonium salt (ABTS), 2,2,-azobis-(2-methylpropionamide) dihydrochloride (AAPH), fluorescein (C_20_H_1_0Na_2_O_5_), and 2,4,6-tripyridyl-*s*-triazine (TPTZ) were purchased from Sigma–Aldrich Company Ltd. (Gillingham, UK). Folin-Ciocalteu reagent, absolute ethanol, aluminum oxide, ferric chloride (FeCl_3_), ammonium thiocyanate (NH_4_SCN), anhydrous sodium carbonate, and Tween 20 were of analytical grade, from Panreac (Barcelona, Spain).

### 2.3. Extraction

Air-dried and finely ground perilla was weighed (4 g) and extracted with 60 mL of ethanol-water mixture at 50:50 (v/v). The mixture was stirred continuously for 24 h at 4 °C. After that, all samples were centrifuged (Sigma 6K10, Osterode am Harz, Germany). Part of the supernatant was used to determine the antiradical capacity. The volume of the remaining supernatant was measured and the solution was evaporated, frozen at −80 °C for 24 h, and lyophilized for 3 days. Samples were then weighed and kept protected from light in a desiccator until used to prepare an oil-in-water emulsion system.

### 2.4. Total Phenol and Flavonoid Content

Total polyphenol content (TPC) of extracts was determined by colorimetric spectrophotometry following the Folin-Ciocalteu method [20], slightly modified and adapted for microplates. Samples were taken from the extract solutions, diluted 1:30 (v:v) and Folin-Ciocalteu reagent (4% by volume), 20% sodium carbonate solution (30.8% by volume), and Milli-Q water were added. Samples were well mixed and left in the dark for 1 h. The absorbance was measured at 725 nm using a UV-vis spectrophotometer (Fluostrar Omega, Perkin-Elmer, Paris, France) and the results were expressed in gallic acid equivalents, GAE, using a gallic acid standard curve (10–70 µM).

Total flavonoid content (TFC) of perilla extracts was measured according to the method of Jia *et al*. [21]. Each sample (500 µL) was mixed with 5% NaNO_2_ (75 µL), and then left to stand for 5 min at room temperature. The mixture was sequentially mixed with 150 µL 10% AlCl_3_, 500 µL 1 M NaOH and 275 µL distilled water. The absorbance at 510 nm was measured using spectrophotometer UV-4201/20 (Zuzi, AuxiLab, S.L., Navarra, Spain). Values were determined from a calibration curve prepared with catechin (ranging from 6 to 60 mg/L) and expressed as mg of catechin equivalent per gram of dry weight (CE/g DW).

### 2.5. Antioxidant Capacity Determination

#### 2.5.1. ABTS Assay

The first method used was the 2,2′-azino-bis-3-ethylbenzo-thiazoline-6-sulphonic acid (ABTS) discoloration assay [22]. The assay is based on the ability of an antioxidative compound to quench the ABTS^+^ radical relative to that of a reference antioxidant such as Trolox. A stock solution of ABTS radical cation was prepared by mixing ABTS solution with a potassium persulfate solution at 7 mM and 2.45 mM final concentration, respectively. The mixture was maintained in the dark at room temperature for 16 h before use. The working ABTS^+^ solution was produced by dilution of the stock solution in 10 mM PBS (pH 7.4) incubated at 30 °C to achieve an absorbance value of 0.7 (±0.02) at 734 nm. An aliquot of 20 µL of diluted extract was added to ABTS^+^ radical working solution (180 µL). For the blank and standard curve, 20 µL of PBS or Trolox solution were used, respectively. Absorbance was measured by means of a UV-vis spectrophotometer (Fluostar Omega, Perkin-Elmer, Paris, France) at 734 nm and percent inhibition was calculated as per Skowyra *et al*. [5]. The radical-scavenging capacity of extracts was quantified as mg of Trolox equivalent per gram of dry weight (TE/g DW).

#### 2.5.2. The Oxygen Radical Absorbance Capacity (ORAC) Assay

The oxygen radical absorbance capacity (ORAC) method was adapted from Ou *et al*. [23]. The assay was performed with an automated microplate reader and 96-well plates. Diluted extract (40 µL) was transferred by pipette into each well and then 120 µL of 1.34 µM fluorescein working solution in phosphate buffer (13.3 mM) at 37 °C were added to each sample. The plate was placed in a spectrophotometer (Fluostar Omega, Perkin-Elmer, Paris, France) and incubated at 37 °C. The initial fluorescence was recorded at an excitation wavelength of 485 nm and an emission wavelength of 535 nm. 2,2′-Azobis(2-metylpropionamide) dihydrochloride (AAPH, 40 µL, 30 mM) was then added to each sample well and the fluorescence was measured immediately and every 2 min thereafter for 120 min. For the calibration curve, solutions of Trolox were prepared in the range of 8–58 µM. The ORAC value for each extract was calculated using a regression equation relating Trolox concentration and the net area under the fluorescence decay curve. Results are expressed as mg of Trolox equivalents per gram of dry weight (TE/g DW).

#### 2.5.3. FRAP Assay

The FRAP assay was performed as described by Benzie & Strain [24] with some modifications. The FRAP reagent was prepared with acetate buffer (300 mM, pH 3.6), TPTZ (10 mM in HCl, 40 mM) and FeCl_3_ (20 mM). The proportions were 10:1:1 (v:v:v), respectively. A suitable dilution of the extract was added to the FRAP reagent (1:30, v:v) and incubated at 37 °C. The assay was performed by means of an automated microplate reader (Fluostar Omega, Perkin-Elmer, Paris, France) with 96-well plates. The absorbance at 593 nm at time zero and after 4 min was recorded. The analysis was performed in triplicate and values were determined from a calibration curve of Trolox (ranging from 2.5 to 33 µM). The results are expressed as mg of Trolox equivalent per gram of dry weight (mg TE/g DW).

### 2.6. Determination of Cinnamic Acid Derivatives by High-Performance Liquid Chromatography (HPLC)

HPLC analyses of the perilla extracts were carried out using an Acquity UPLC System (Waters, Milford, MA, USA) with photodiode array (PDA) detector. Perilla extracts (10 µL) were injected onto an analytical C_18_ column (Symmetry, 5 µm, 3.9 × 150 mm, Waters) at 25 °C. The mobile phase was composed of 0.5% formic acid (v/v) in acetonitrile (eluent A) and 0.5% formic acid (v/v) in water (eluent B). The gradient program was as follows: 10% A (20 min), 35% A (4 min), 10% A (6 min). Total run time was 30 min. The absorbance at 330 nm was measured to detect the cinnamic acid derivatives (caffeic acid and rosmarinic acid). The standard was identified by its retention time and its concentration was calculated by comparing the peak area of samples with that of the standard. Standard solutions with concentrations ranging from 10 to 100 ppm were then prepared by diluting the stock standard solution with water. The perilla extracts were filtered through a 0.45 µm filter for HPLC analysis.

### 2.7. Oil-in-Water Emulsion System

#### 2.7.1. Removal of Tocopherols from Sunflower Oil

Tocopherols were removed from sunflower oil by column chromatography using activated alumina, as described by Yoshida *et al*. [25]. The oil was stored at −80 °C prior to emulsion preparation (up to 2 days). The fatty acid composition of the filtered sunflower oil is shown in Table 1. The fatty acid composition was determined using a method based on that of Conde *et al*. [26]. The sunflower oil used contained linoleic acid (52.38%) and oleic acid (34.51%) as the main unsaturated fatty acids.

#### 2.7.2. Preparation of Emulsions and Storage Conditions

Oil-in-water emulsions were prepared with 1% of Tween 20 as emulsifier and 10% of sunflower oil (2.7.1). Emulsions were prepared by dropwise addition of oil to the water phase, with sonication using a UP200S ultrasonic (Hielscher Ultrasonics GmbH, Teltow, Germany) during cooling in an ice bath for 10 min. It was necessary to repeat sonication 7 times (7 × 10 min) to have enough volume of emulsion. Freeze-dried powder of the perilla extract was redissolved in ethanol 50% (v/v) and added directly to the emulsion and homogenized, obtaining final concentrations of 80 and 320 ppm (C1 and C2, respectively). For the negative control, no extract was added, and the positive controls were prepared with Trolox (40 ppm) and BHA (20 ppm) dissolved in ethanol.

All emulsions were stored in triplicate in 60 mL amber bottles in the dark, with constant elliptical movement and allowed to oxidize at 32 ± 1 °C for 30 days.

**Table 1:** Fatty acid composition of sunflower oil.

Fatty acid name	Numerical symbol	Amount (%)
**Saturated**		**12.79**
Palmitic acid	C16:0	6.99 ± 0.08
Stearic acid	C18:0	4.16 ± 0.04
Arachidic acid	C20:0	0.33 ± 0.01
Behenic acid	C22:0	0.96 ± 0.02
Lignoceric acid	C24:0	0.35 ± 0.02
**Unsaturated**		**87.21**
Oleic acid	C18:1 (*n*-9)	34.51 ± 0.11
Eicosenoic acid	C20:1 (*n*-9)	0.32 ± 0.03
Linolenic acid	C18:2 (*n*-6)	52.38 ± 0.23

#### 2.7.3. Measurement of Primary Oxidation by Peroxide Value (PV) and pH

Peroxide value (PV) was measured periodically (every 2 or 3 days, the time of storage) using aliquots of 0.05–0.1 g of each sample and determined by the ferric thiocyanate method [27], after calibrating the procedure with a series of oxidized oil samples analyzed by the AOCS Official Method Cd 8-53 [28].

The pH of the samples was measured (pH-meter GLP21, Criston Instruments, Barcelona, Spain) as a parameter to investigate its correlation with PV.

#### 2.7.4. Measurement of Secondary Oxidation by TBARs and Hexanal Methods

The thiobarbituric acid reactive substances (TBARs) assay was performed as described by Maqsood and Benjakul [29], with some modifications. One milliliter of oil-in-water emulsion sample was mixed with a TBARs solution containing 0.375% thiobarbituric acid and 15% trichloroacetic acid in 0.25 N HCl solution (5 mL). The samples were placed immediately in an ultrasonic bath (Prolabo brand equipment) for 5 min and heated in a water bath (95 °C) for 10 min. The mixture was centrifuged (Sigma 3K30, Sigma Laborzentifugen GmbH, Osterode am Harz, Germany) at room temperature at 4000 rpm for 10 min. The absorbance of the supernatants was measured at 532 nm (Spectrophotomter UV-4201/20, Zuzi, Navarra, Spain). The TBARs values were expressed as mg of malondialdehyde (MDA) per kg of emulsion calculated using 1,1,3,3-tetraethoxypropane (Sigma-Aldrich, St. Louis, MO, USA) as the standard.

Hexanal was measured according to Waraho *et al*. [2], with some modifications, using a TRACE gas chromatography equipped with mass spectrometry MS DSQI (ThermoFisher Scientific, Waltham, MA, USA) and TRIPLUS auto-injector. 1 mL of emulsion was weighted into a head space vial and equilibrated at 60 °C for 30 min. Aliquots (1 mL) of the head space were injected onto a DB-624 column (60 m × 0.32 mm × 1.8 µm). Initially, the oven temperature was set to 60 °C, maintained at this value for 2 min, then raised up to 220 °C at 8 °C/min and maintained for 5 min. The injection port was operated in the split mode. The carrier gas was helium at flow rate of 1.8 mL/min. A flame ionization detector was used at a temperature of 260 °C. Hexanal concentrations were determined from peak areas using a standard curve prepared with hexanal standard solutions.

### 2.8. Statistical Analysis

TPC, TFC, ABTS^+^, ORAC, and FRAP measurements were performed in triplicate on triplicate samples. PV, TBARs, and hexanal measurements were performed once on triplicate samples.

Mean values for different parameters were calculated and compared by analysis of variance (one-way ANOVA) using commercial software (Minitab 16). Moreover, statistical differences between mean values were identified at the 95% of confidence level (*p* < 0.05). Person’s correlation analysis was performed using the same statistical package.

## 3. Results and Discussion

### 3.1. Phenolic and Flavonoid Content of Extract

The total polyphenols and flavonoids in the ethanolic extracts of *P. frutescens* leaves and stalks are shown in Table 2. The perilla extract contained 22.67 ± 0.52 mg gallic acid equivalent (GAE)/g DW and 2.90 ± 0.07 mg catechin equivalent (CE)/g DW. Hong and Kim [17] reported a similar content in perilla leaves (12.15 mg GAE/g DW and 7.23 mg rutin equivalent (RE)/g DW, respectively) using 70% ethanol in refluxed extraction for 24 h. Similarly, Kee *et al*. [30] found a value of 27.10 mg GAE/g DW after aqueous extraction. However, studies involving methanol extraction of perilla leaves have reported highest values than those obtained in the present study, in the range of 0.7–1.1 g GAE/100 g fresh weight (FW), using a mixture of water-methanol-formic acid (15:80:5) [31]. Lin *et al*. [15] also studied the extraction of phenolics and flavonoids from perilla leaves and stalks separately and the methanolic extract of stalk had higher polyphenol and flavonoids content than that of leaves (137.40 mg GAE/L and 205.75 mg RE/L, respectively). Consequently, the extraction method and the solvent used play a key role in the extraction of polyphenols and flavonoids from plant material. Likewise, Hong *et al*. [32] studied phenolic-enriched fraction from *P. frutescens* and the highest total phenolic and flavonoid level was detected in the ethyl acetate fraction from the water extract (373.92 mg GAE/g fraction and 86.63 mg RE/g fraction, respectively). Whatever the considered extraction method or solvent, perilla was shown to have polyphenol level like typical culinary herbs from the same family (*Lamiaceae*), such as basil [33], spearmint [30], marjoram, and salvia [34].

**Table 2:** Polyphenol and flavonoid content and antioxidant activity of perilla extract.

Method	Amount detected ^a^
Total phenol content (mg GAE/g DW)	22.67 ± 0.52
Total flavonoid content (mg CE/g DW)	2.90 ± 0.07
ABTS (mg TE/g DW)	65.03 ± 2.98
ORAC (mg TE/g DW)	179.60 ± 6.02
FRAP (mg TE/g DW)	44.46 ± 1.55

^a^ Values are mean ± standard deviation (*n* = 3).

### 3.2. *In Vitro* Antioxidant Activity of Extract

Antioxidant activity of the extract from *P. frutescens* was assessed by three different methods: ABTS^+^, ORAC, and FRAP (Table 2). The use of several methods provides more comprehensive information about the antioxidant properties of the original product [35]. An ABTS^+^ value of 65.03 ± 2.98 mg TE/g DW, an ORAC value of 179.69 mg TE/g DW, and a FRAP value of 44.46 mg TE/g DW were measured in the ethanolic leaf and stalk extract. Similarly, Muller-Waldeck [31] found the ABTS^+^ value of 0.8–1.3 g TE/100 g fresh weight (FW) in perilla leaves using 80% methanol with 5% formic acid. Lin *et al*. [15] reported that a scavenging abilities of the methanolic extracts of stalk and leaf from *P. frutescens* at 1.5–25 µg/mL on DPPH radicals were in the range of 18.7%–91.0% and 6.7%–63.1%, respectively. Meng *et al*. [36] also found an activity of 114–167 µmol TE/100 mL of aqueous perilla extract as assessed via DPPH assay. Hong *et al*. [32] described antiradical activity (DPPH) and reducing power of the phenolic-enriched fractions from *P. frutescens*, finding strong reducing power and effective radical scavenging activity (89.48%–90.74%) of the ethyl acetate fractions of methanolic extracts. They also reported the antioxidant activity (in β-carotene/linoleic acid system) of the phenolic-enriched fractions from perilla leaves, finding that at 500 µg/mL chloroform fractions of 70% ethanol extract presented remarkable antioxidant abilities in the linoleic acid emulsion system. Tawaha *et al*. [34] assessed the antioxidant activity of selected plant species such as rosemary, marjoram and salvia by ABTS^+^ method, obtaining in all cases lower values (of the order of µmol TE/g DW) than those found for perilla in this study. In addition, Hossain *et al*. [33] reported that the ORAC values for basil and parsley were 17.57 and 13.25 g TE/100 g DW, respectively. This value is much lower than those found in the current study.

### 3.3. Quantitative Analysis of Cinnamic Acid Derivatives

Lately, phenolic compounds, such as rosmarinic acid or caffeic acid have aroused increasing interest due to their antioxidant activity, which improves the stability of lipid-containing foods [26,37] and their possible beneficial effects on human health [38]. The concentrations of rosmarinic acid (2.29 ± 0.09 mg/g DW) and caffeic acid (0.51 ± 0.02 mg/g DW) were compared with those reported in the literature (Table 3).

**Table 3:** Content of rosmarinic acid and caffeic acid in the perilla extracts (mg/g DW).

Rosmarinic acid	Caffeic acid	Solvent	Place of cultivation	Reference
39.5	ND	Water:acetone:hydrochloric (20:80:1)	Japan	Natsume *et al*. [39]
3.4–10	0.05–1.2	Water with 0.01 M H_2_SO_4_	China and Japan	Meng *et al*. [36]
0.21–3.76	ND	70% EtOH	Geochang, Korea	Hong and Kim [17]
51.37–155.50	ND	MeOH with ethyl acetate fraction	Geochang, Korea	Hong *et al*. [32]
29.28–54.76	1.09–3.86	MeOH with 1% TFA	Yeongnam, Korea	Kang and Lee [40]
26.84	1.32	Water at 100 °C	Miryang, Korea	Yang *et al*. [38]
2.29	0.51	50% EtOH	Spain	This paper

It is well known that the different extraction conditions lead to different amount of polyphenols in plant extracts. Although many studies have reported the content of cinnamic acid derivatives in *P. frutescens*, researches on the content of the main phenolic compounds in perilla cultivated in Europe has not been measured yet.

The utilization of perilla opens interesting possibilities for the development of functional foods not only in Asian countries, but also in Europe. Moreover, the content of cinnamic acid derivatives in perilla extract was higher or similar to those reported by Lee [41] in the dried plants (*Lamiaceae*), such as basil, marjoram, oregano, rosemary, or thyme, which are common culinary herbs.

### 3.4. Antioxidant Activity of Extracts in Model Emulsion System

In addition to their effects on human health upon direct ingestion, one of the main applications of antioxidant extracts is their use in the food industry to avoid or delay oxidation of perishable systems [42]. In this study, this technological ability has been assessed in oil-in-water emulsions as a model food used to test the 50% ethanol extract of perilla. The oxidation was periodically followed by measurement of peroxide value (as indicator of primary oxidation products) and TBARs value and hexanal content (as indicator of secondary oxidation products) during storage at 32 ± 1 °C for 30 days. In addition the change in pH was monitored, as pH tends to fall during oxidation.

**Figure 1:**
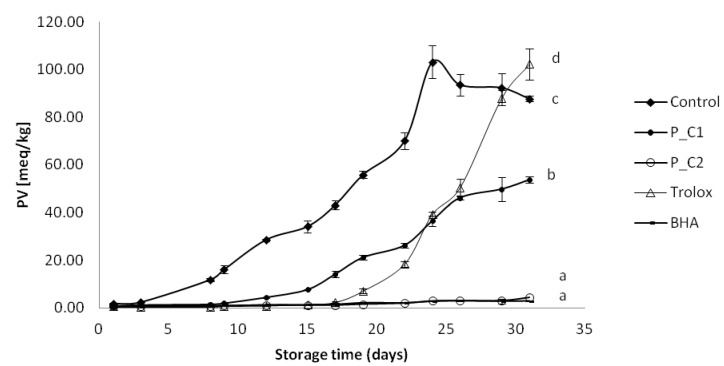
Evolution of primary oxidation (peroxide value) in model food system (O/W emulsion 10% of oil) with different concentration of perilla ethanolic extracts (C1: 80 ppm and C2: 320 ppm).

^a–d^ Values with different letters at 30th day of storage are significantly different (*p* < 0.05).

Peroxide values in the oil emulsions increased significantly faster in the sample without any antioxidant addition (Figure 1), reaching 10 meq hydroperoxides/kg of emulsion (this value is the allowed limit for products containing edible fats) after six days. The next samples to reach this level of deterioration were P_C1 perilla at 80 ppm (after 15 days) and Trolox at 40 ppm (after 19 days). Other samples: P_C2 and BHA were stable until the end of the experiment (after 30 days, PV was <10 meq/kg). Perilla extract at 320 ppm (P_C2) was as effective as BHA at 20 ppm in preventing the oxidation in emulsions during storage. The mean peroxide value recorded for control emulsions after 24 days, was more than twice the value obtained for emulsions containing the perilla extract at 80 ppm and Trolox at 40 ppm (control: 103.17 meq/kg; perilla C1: 36.55 meq/kg; Trolox: 39.42 meq/kg). Kiokias *et al*. [43] reported peroxide values between 45.60 and 51.15 meq/kg after two months in 10% sunflower oil-in-water emulsions with 2 g/L of different carotenoids including β-carotene, lycopene, paprika, lutein, and bixin. Although the time needed to reach these values was remarkably shorter in this study, the concentration of antioxidant extracts was much lower as well (80 and 320 ppm). Kiokias and Varzakas [44] reported that a 10% cottonseed oil-in-water emulsion with quercetin at 1.5 mmol/kgr took 2.7 days at 60 °C to reach a PV of 67.07 meq/kg. In addition, Maisuthisakul *et al*. [45] reported that a 10% oil-in-water emulsion with 100 mg/kg teaw (*Cratoxylum formosum* Dyer) extract took 4.55 days at 60 °C to reach a PV of 50 meq/kg. In this work, the emulsion containing 80 ppm of perilla extract exceeded this value at the 30th day at 32 °C. Ramful *et al*. [9] found that *Eugenia pollicina* leaf extract at a concentration of 0.02% was also effective in slowing down hydroperoxide formation in soybean oil emulsion during 13 days of storage at 40 °C. Roedig-Penman *et al*. [46] reported that tea extracts added to sunflower oil-in-water emulsion were very effective in their stabilization, the tea extract (0.03%) being similar to BHT (0.02%) and taking 40 days of storage at 30 °C to reach a PV of 30 meq/kg. In the same study the rosemary extract (0.03%) in the early stages of storage under these conditions had moderate antioxidant activity up to PV of 20 meq/kg.

**Figure 2:**
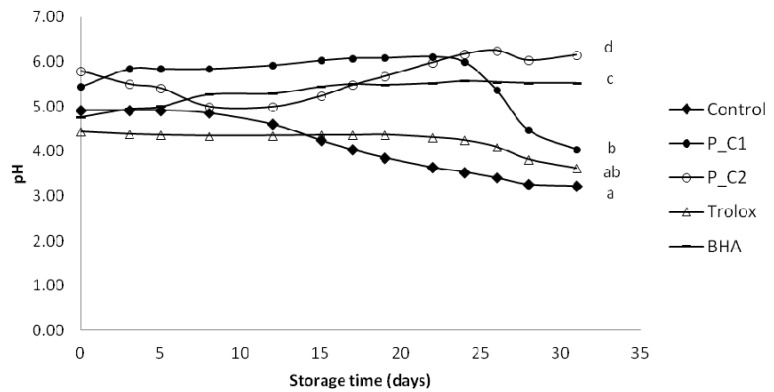
Evolution of pH in model food system (O/W emulsion 10% of oil) with different concentration of perilla ethanolic extracts (C1: 80 ppm and C2: 320 ppm).

^a–d^ Values with different letters at 30th day of storage are significantly different (*p* < 0.05).

Hydroperoxides are the main primary products of lipid oxidation, but they are highly unstable and easily break down into secondary compounds, resulting in the appearance of aldehydes, ketones, epoxides, or organic acids, which may lead to changes in the pH [46]. In addition, since it is known that many antioxidant molecules are less effective when the pH is low [47], this parameter was also measured as a potential indicator of oil-in-water emulsions oxidation. From an initial average value of 5.12, the samples without any antioxidant addition and with Trolox tended to stabilize their pH at 3.22 and 3.62, respectively after 30 days (Figure 2). In the P_C1, P_C2 and BHA samples the pH slowly increased during storage, but in P_C1 pH it decreased rapidly after 24 days, reaching the value of 4.05. Observing this relationship confirmed that the pH fell as PV increased. Gallego *et al*. [12] reported that following the order of primary oxidation, the pH showed a decline (from 6 to 3) proportional to the rate of oxidation in oil-in-water emulsion with 100 ppm of different extracts from rosemary, thyme, and lavender. Sorensen *et al*. [48] also reported that lipid oxidation increased when pH was decreased from 6 to 3 in a 10% oil-in-water emulsion. Moreover, Zhou *et al*. [49] found that the pH appeared to play a significant role in controlling the net antioxidant and pro-oxidant capacity of polyphenols in lipid model systems. The addition of (−)-epigallocatechin-3-gallate (EGCG) at 5–100 µM to food emulsions was observed to exhibit pro-oxidant activity in low pH (pH 2–4). On the other hand at higher pH values studied (pH 5–7), lower levels of primary and secondary oxidation products were detected in samples with 25–500 µM EGCG.

**Figure 3:**
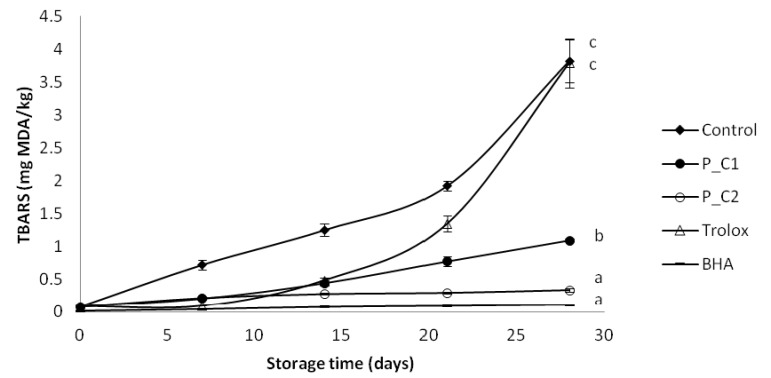
Evolution of secondary oxidation (TBARs) in model food system (O/W emulsion 10% of oil) with different concentration of perilla ethanolic extracts (C1: 80 ppm and C2: 320 ppm).

^a–c^ Values with different letters at 30th day of storage are significantly different (*p* < 0.05).

**Figure 4:**
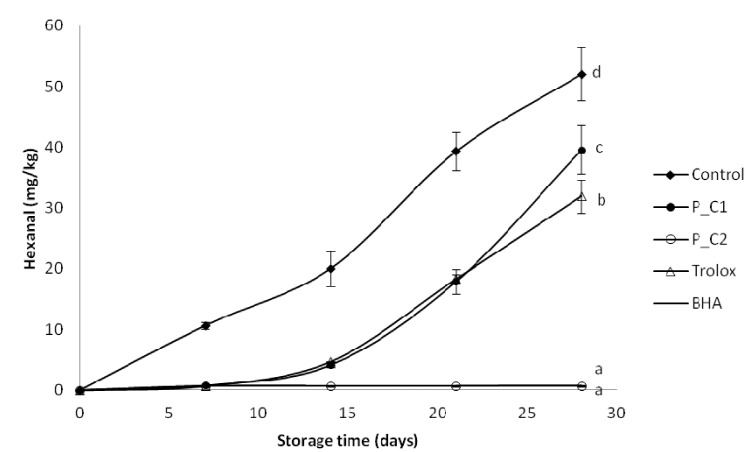
Evolution of secondary oxidation (hexanal content) in model food system (O/W emulsion 10% of oil) with different concentration of perilla ethanolic extracts (C1: 80 ppm and C2: 320 ppm).

^a–d^ Values with different letters at 30th day of storage are significantly different (*p* < 0.05).

In the secondary oxidation stage, volatile compounds (e.g., alcohols and aldehydes) are formed by the decomposition of lipid hydroperoxides. In particular, volatile aldehydes have great importance as an indicator of oxidation due to their considerable contribution to the aroma and flavor deterioration of the final product [50]. Secondary oxidation products in the emulsions were monitored by measurement of the TBARs (Figure 3) and the hexanal content (Figure 4). After four weeks, TBARs values of emulsions containing perilla extract and BHA were lower than that of the control (3.82 mg MDA/kg) and the Trolox-containing sample (3.57 mg MDA/kg). BHA was the most effective antioxidant followed by perilla extract P_C2 and P_C1. Garcia-Iñiguez *et al*. [13] reported that a lyophilized aqueous extract of *Melissa officinalis* (lemon balm) at 620.6 ppm was as efficient as BHA at 200 ppm in controlling the TBARs formation in oil-in-water emulsions made with a mixture of algae and lineseed oils upon storage during 15 days at 20 °C. In addition, Poyato *et al*. [14] reported that in olive oil-in-water emulsions after 48 h of storage at 65 °C, TBARs value was stable and low, with no differences between lyophilized aqueous extract of lemon balm (477 ppm) and BHA (200 ppm). Dimakou and Oreopoulou [51] found that polar (paprika, marigold, bixin) and hydrophobic (β-carotene, lycopene) carotenoids exerted antioxidant effect measured by TBARs test during thermal accelerated autoxidation (60 °C) of sunflower oil-in-water emulsions, stabilized by Tween 20.

Similar to TBARs, the results of hexanal content after four weeks of storage showed that BHA and perilla extract (P_C2: 320 ppm) were the most effective antioxidant, followed by Trolox and P_C1. The protective effect of *P. frutescens* should be attributed to its content of the well-recognized antioxidant, like hydroxycinnamic acids and possibly other polyphenols. In the literature, other authors have described the antioxidant effect of caffeic acid and rosmarinic acid in model food emulsions. Caffeic acid (5 mmol/kg emulsion) showed good antioxidant properties in both 30% sunflower oil-in-water and 20% water-in-sunflower oil emulsions (pH 5.4) during storage at 50 °C [50]. In the model corn oil-in-water emulsions rosmarinic acid (50 µM) inhibited hexanal formation during storage at 55 °C for 24 days [1].

In the present study positive correlations between TBARs and hexanal (*R*^2^ = 0.9106) levels and also between PV and both TBARs and hexanal (*R*^2^ = 0.9446 and *R*^2^ = 0.9431, respectively) levels in oil-in-water emulsions were found.

Different extracts of *P. frutescens* have demonstrated free-radical scavenging [17,31,36,40] and antioxidant action in rats [38,52]. These properties of perilla have been associated with cinnamic acid derivatives and other polyphenolic compounds. Caffeic acid (CA) and rosmarinic acid (RA) are the major compounds in *P. frutescens* that showed the hepatoprotective effect against t-BHP-induced oxidative liver damage. *In vitro* and *in vivo* treatments with perilla leave extracts and with combined CA and RA gave almost the same efficacy of liver protection against oxidative stress. *In vivo* treatment of combined CA and RA resulted in a more than proportional increase in antioxidant enzymes and reduced levels of indicators of hepatic toxicity, compared to CA only treatment suggesting that the stronger hepatoprotective effect of perilla is brought about by the combination of CA and RA. In an *in vivo* study, samples pretreated with 1000 mg/kg of body weight (BW) of perilla extract showed no signs of toxicity. After the conversion of the effective dosage in rats into a dose based on the surface area of humans they obtained the perilla extract equivalent in humans of 162 mg/kg BW, which equates to 9.7 g of perilla extract o 205 g of perilla fresh leaves [38].

The concentration of the perilla extract was selected so as not to exceed the non-toxic pharmacological doses. Therefore, the addition of 320 mg/kg of perilla extract to food emulsions would serve a twofold purpose: (i) to support the pharmacological doses of these extracts by using those food emulsion system as vectors; and (ii) to improve the fat stability and, hence, the nutritional properties of those emulsion systems.

## 4. Conclusions

This study showed that the extract of *Perilla frutescens* is a potential source of natural antioxidant to be used as a lipid oxidation inhibitor in food industry. In addition, food emulsions appear to be useful vectors in supplying the daily dosage of *P. frutescens* extract in consumers, which may positively affect their health. Further research into the enrichment of food products with bioactive substances extracted from *P. frutescens* should be conducted because there is still not sufficient knowledge about their activity during food processing, nor about their interactions with other food components.
